# 

**Published:** 1877-10

**Authors:** 


*CONTENTS.*

| | Page |
|---|---|
| Safety of Families | 267 |
| Laughter and Music | 269 |
| Death's Weapons | 270 |
| Avenues of Death | 271 |
| Cure of Corns | 273 |
| Hearty Suppers | 275 |
| The Secret Out | 276 |
| Trials of Life | 278 |
| Home | 279 |
| Marriage in England | 280 |
| The Rest | 282 |
| The Little Boy that Died | 282 |
| The Expression of Dress | 283 |
| Who are Happiest? | 284 |
| Apples | 287 |
| Thinking Brag | 288 |
| Preventing Disease | 289 |
| Greed of Gold | 289 |
| Daring to Do | 291 |
| Bowel Complaints of Children | 292 |

